# Reprogramming the Melanoma Tumor Microenvironment: From Cellular Crosstalk to Cellular Immunotherapy

**DOI:** 10.3390/cancers18203325

**Published:** 2026-10-15

**Authors:** Enar Jumaniyazova, Elena Gantsova, Genrikh Kazarian, Irina Charyeva, Timur Fatkhudinov

**Affiliations:** 1 Research Institute of Molecular and Cellular Medicine, Peoples’ Friendship University of Russia (RUDN University), 6 Miklukho-Maklaya Street, 117198 Moscow, Russia; 2 Avtsyn Research Institute of Human Morphology of FSBSI Petrovsky National Research Centre of Surgery, 3 Tsyurupy Street, 117418 Moscow, Russia

**Keywords:** melanoma, tumor microenvironment, immune cells, immunotherapy, cellular immune therapy, tumor-associated macrophages, tumor-infiltrating lymphocytes

## Abstract

Melanoma is the most aggressive and rapidly metastasizing malignant neoplasm of the skin and mucous membranes, arising from melanocytes of neuroectodermal origin. Despite substantial advances in immune checkpoint inhibitors and targeted therapy, a significant proportion of patients develop primary or acquired resistance, which is largely attributable to the complex interactions between tumor cells and the tumor microenvironment (TME). This review presents a comprehensive analysis of the cellular and molecular components of the melanoma TME and their contribution to immunosuppression, tumor progression, and therapeutic resistance. The role of keratinocytes as the primary niche-forming cells in the epidermis is examined, including their reprogramming during melanomagenesis and their involvement in drug resistance. Particular attention is given to cancer-associated fibroblasts (CAFs), which orchestrate stromal remodeling, promote immune exclusion, and limit the efficacy of immunotherapy. The phenotypic and functional heterogeneity of innate and adaptive immune cells—macrophages, granulocytes, natural killer cells, dendritic cells, T and B lymphocytes—is discussed in the context of their dual role in antitumor immunity and immune escape. A detailed analysis of immune editing mechanisms, including defective antigen recognition, resistance to apoptosis, and the establishment of an immunosuppressive microenvironment, is provided. The review further summarizes the current landscape of cellular immunotherapy for melanoma. Clinically established approaches, including tumor-infiltrating lymphocytes (TILs) and CAR-T cells, are discussed with reference to completed phase II–III clinical trials, whereas emerging strategies such as CAR-macrophages and dendritic cell vaccines are presented as largely preclinical or early-phase investigational platforms. It is concluded that the melanoma TME functions as a unified immunosuppressive network rather than a collection of individual cell populations. Based on currently available comparative clinical evidence, reprogramming this network through the combination of cellular platforms with immune checkpoint inhibitors, stromal modulators, and macrophage-repolarizing agents, supported by personalized assessment of TME composition, represents a rational and actively investigated therapeutic direction.

## Simple Summary

Melanoma remains the most aggressive form of skin cancer, and despite significant advances in immunotherapy and targeted therapy, resistance to treatment continues to develop in a substantial proportion of patients. A growing body of evidence indicates that the tumor microenvironment (TME)—the complex network of keratinocytes, fibroblasts, and immune cells surrounding the tumor—plays a central role in this resistance. This review examines how distinct cellular components of the melanoma TME interact to form a self-sustaining immunosuppressive network that promotes tumor progression and limits the efficacy of current therapeutic approaches. Particular attention is given to the translation of these insights into cellular immunotherapy platforms, including tumor-infiltrating lymphocytes, CAR-T cells, CAR-macrophages, and dendritic cell vaccines. Strategies aimed at reprogramming the TME rather than targeting tumor cells alone are discussed. It is concluded that personalized assessment of TME composition, combined with the integration of cellular platforms and immune checkpoint inhibitors, may represent a rational and actively investigated direction for improving clinical outcomes in melanoma.

## 1. Introduction

Melanoma is a malignant neoplasm of the skin and mucous membranes that arises from melanocytes, cells of neuroectodermal origin [1,2]. Among all cutaneous malignancies, melanoma is the most aggressive and rapidly metastasizing tumor. In 2022, an estimated 331,722 new cases of cutaneous melanoma were diagnosed worldwide, and approximately 58,667 deaths were attributed to this disease [3]. The global incidence of cutaneous malignant melanoma continues to rise, increasing by approximately 2.6% per year [4]. The epidemiological patterns of melanoma prevalence and incidence are highly variable and depend on factors such as racial background and geographic region, including ozone layer integrity and climatic characteristics. Thus, in Western countries, cutaneous melanoma predominantly affects individuals of European ancestry, and ultraviolet (UV) radiation exposure has been recognized as the primary etiological factor [5]. In contrast, in Asia and Africa, acral melanomas predominantly develop among non-European populations, accounting for nearly 60% of all diagnosed melanoma cases in these regions [6,7]. Among proposed risk factors for acral melanoma, chronic mechanical stress has gained increasing attention. Preclinical studies suggest a mechanistic basis: mechanical loading can induce nuclear envelope rupture and DNA damage via YAP activation in melanoma cells, and in vitro compression promotes invasive behavior [8]. Epidemiological data indicate that acral melanomas on the foot more frequently arise in weight-bearing areas, although this association is correlative. The role of acute trauma remains controversial, with positive associations reported primarily in Asian populations, while studies from Western countries have not consistently confirmed this relationship. Therefore, mechanical stress should be considered a plausible but not definitively established risk factor for acral melanoma [9].

For cutaneous melanoma, UV radiation is recognized as the key etiological factor, initiating a cascade of molecular damage in melanocytes, particularly through direct DNA damage [10]. It has been established that in 2022, more than 80% of cutaneous melanoma cases in the global population were attributable to ultraviolet radiation exposure [11]. Previously, UV-B was considered the primary inducer of melanoma; however, in-depth studies of photochemical DNA damage and the consequences of ozone depletion have led to a revision of this view: both UV ranges contribute pathogenetically, and thinning of the ozone layer amplifies the cumulative mutagenic burden on melanocytes [12]. It has been shown that a 1% decrease in atmospheric ozone increases the flux of biologically active UV-B at the Earth’s surface by 1.5–2%, thereby enhancing the mutagenic impact on melanocytes [13]. UV-B is directly absorbed by the DNA of keratinocytes and melanocytes, inducing the formation of cyclobutane pyrimidine dimers (CPDs) and pyrimidine-(6-4)-pyrimidone photoproducts (6-4PPs). These adducts are the principal cause of the characteristic mutation signature (signature 7 according to the COSMIC catalog): C→T and CC→TT transitions detected in melanoma driver genes (BRAF, NRAS, TP53, and the TERT promoter) [14]. UV-A also generates CPDs, but not directly; rather, it does so through melanin excitation with the generation of reactive oxygen species in the dark phase (“dark CPDs”). Thus, melanin, which is intended to protect the nucleus, paradoxically acts as a photosensitizer [15].

In addition to natural UV radiation, artificial irradiation—primarily from tanning bed use—contributes substantially to melanoma development [16]. Indeed, individuals who use artificial tanning before the age of 35 have a 75% increased lifetime risk of developing melanoma. The risk increases with years of use, number of sessions, and total hours of UV exposure [17,18,19,20].

Other risk factors for melanoma include chronic mechanical or chemical skin irritation, low pigment content, and genetic predisposition, particularly familial atypical mole syndrome [20]. Since cutaneous melanoma in most cases develops at the site of pre-existing nevi, often as a result of their mechanical trauma, the presence of multiple nevi is also considered an important risk factor [5]. Alongside external and hereditary factors, sex-specific characteristics play an important role, determining differences in melanoma incidence, anatomical localization, and outcomes (Table 1).

The vast majority of melanomas are sporadic, whereas approximately 5–12% of melanoma patients have a family history of the disease [32,33]. It should be emphasized that melanoma is among the malignant neoplasms with the highest mutational burden. In familial melanoma, mutations are frequently identified in the *CDKN2A* gene, which encodes the p14ARF and p16INK4a proteins—key tumor suppressors that regulate the G1 cell cycle checkpoint and stabilize p53 expression [32,33]. In sporadic melanomas, the majority of mutations affect genes encoding components of the MAPK (mitogen-activated protein kinase) signaling pathway, which is responsible for integrating extracellular signals, such as growth factors, with the regulation of intracellular processes including growth, cell division, differentiation, and apoptosis [34]. According to current estimates, mutations in MAPK pathway genes are detected in approximately 70% of melanoma cases. The consequence of such mutations is constitutive activation of the signaling cascade, leading to uncontrolled proliferation of cancer cells [35,36].

Currently, the primary treatment for early-stage melanoma is surgical excision, including wide local excision of the primary lesion and sentinel lymph node biopsy or regional lymphadenectomy, and the majority of early-stage melanomas are curable. For patients with metastatic disease, surgery alone is not curative, and the next step is systemic anticancer therapy [37]. Various forms of immunotherapy are of particular importance, given that the five-year survival rate for patients with metastatic melanoma is only approximately 25% [38]. Although immune checkpoint inhibitors and targeted therapy have substantially improved outcomes in metastatic melanoma, a significant proportion of patients still develop resistance or experience intolerance to full-dose therapy, necessitating the search for additional strategies based on modulation of the immune system [39,40]. Therapy-resistant melanoma remains a major clinical challenge despite advances in immune checkpoint inhibitors and targeted therapy, as most patients eventually develop primary or acquired resistance and proceed through a succession of toxic lines of drug treatment with time-limited efficacy. This resistance reflects not only the intrinsic ability of tumor cells to adapt or become more aggressive during treatment; the TME also plays a critical role [41,42]. Indeed, melanoma resistance has been shown to be associated with T cell dysfunction as well as a profound quantitative deficiency of tumor-reactive lymphocytes, exacerbated by myeloid cell- and regulatory T cell-mediated immunosuppression, impaired antigen presentation, and tumor dedifferentiation programs that create a “cold” TME [43]. Thus, the limited efficacy of current therapeutic approaches is largely attributable to the complex interactions between tumor cells and their microenvironment, which actively participates in establishing an immunosuppressive milieu and driving resistance. Accordingly, increasing attention is being devoted to the study of the cellular and molecular components of the melanoma TME as potential therapeutic targets.

A review of the role of cellular components of the melanoma microenvironment is best conducted in accordance with the stages of tumor progression. At the initial stages of melanomagenesis, key events unfold in the epidermis, where keratinocytes form the immediate microenvironment of melanocytes and are the first to engage in intercellular interactions with transformed cells. As the tumor invades the dermis, the regulatory center shifts toward fibroblasts and immune cells, reflecting the dynamic remodeling of the TME.

This review differs from previously published reviews on melanoma TME and cellular therapies, which typically focus either on immune cell populations or on individual therapeutic platforms. Here, we integrate non-immune TME components (keratinocytes, CAFs) with innate and adaptive immune cells and systematically link these mechanisms to the rationale for cellular immunotherapy in melanoma. Based on the recent literature (2020–2025), this integrated perspective highlights TME-oriented targets that may improve the efficacy of TIL, CAR-T, TCR-T, CAR-NK and DC-based approaches and help overcome therapy resistance.

## 2. Keratinocytes: Active Participants in Melanomagenesis

The most abundant cell population in the melanoma TME is keratinocytes, which under physiological conditions are in close contact with melanocytes and participate in the regulation of their proliferation, differentiation, and melanogenesis [44,45,46]. Keratinocytes contribute to the formation of a permissive niche that may facilitate melanoma initiation. UV radiation passing through the thinned ozone layer induces a massive release of stem cell factor (SCF) and endothelin-1 from keratinocytes—key paracrine mitogens for melanocytes [47]. The interaction between keratinocytes and emerging melanoma cells appears to be dynamic and changes rapidly at the tumor initiation stage, when the keratinocyte gradually transitions from a “gatekeeper” to an “accomplice” [48]. At the early stages of melanomagenesis, keratinocytes can act as a restraining factor—through intercellular contacts and paracrine signals, they are capable of suppressing the proliferation and migration of atypical cells. Keratinocytes secrete growth-inhibitory factors, including TGF-β, which at early stages acts as a potent tumor suppressor [49], but at later stages promotes invasion. E-cadherin-mediated adhesion between keratinocytes and melanocytes transmits signals that limit proliferation and invasive potential. This mechanism is critical during the radial growth phase, when the tumor spreads horizontally but does not penetrate deeply. Melanoma cells actively downregulate E-cadherin expression through the activation of transcriptional repressors (Snail, Slug, Zeb1). This releases β-catenin from the membrane complex, allowing it to translocate to the nucleus and activate proliferative programs. Simultaneously, melanoma cells begin to express N-cadherin (enabling interactions with fibroblasts and endothelium) [50]—a critical step in acquiring invasive capacity and transitioning to the vertical growth phase.

Keratinocytes adjacent to melanoma begin to produce a “cocktail” of pro-inflammatory factors that promote tumor growth: IL-1α and IL-6, which stimulate melanoma cell proliferation; TNF-α, which activates the NF-κB signaling pathway in the tumor, enhancing its survival [51]. In addition, “switched” keratinocytes receiving signals from tumor cells begin to actively produce vascular endothelial growth factor (VEGF) [45], which initiates angiogenesis, and matrix metalloproteinases 1 and 9 (MMP-1, MMP-9), which degrade type IV collagen—one of the key structural components of the basement membrane—thereby facilitating melanoma cell invasion into the dermis [52,53].

Golan et al. demonstrated that differentiated keratinocytes contribute to melanoma progression by activating the Notch signaling pathway in tumor cells [54]. The Notch pathway is known to regulate skin homeostasis, melanocyte stem cell maintenance, and the development of cutaneous malignancies. Early evidence has shown elevated Notch receptor expression and constitutive pathway activation in human melanoma lesions [55,56]. Moreover, through crosstalk with other signaling cascades, including ERBB, Nodal, and Wnt, Notch signaling controls several key aspects of melanoma pathogenesis, notably tumor cell survival and proliferation [57,58]. Activated Notch suppresses MITF function, which ultimately relieves the repression of genes (such as miR-222/221) that stimulate invasion and metastasis. In other words, keratinocytes indirectly drive the tumor toward more aggressive behavior [54].

**Figure 1:**
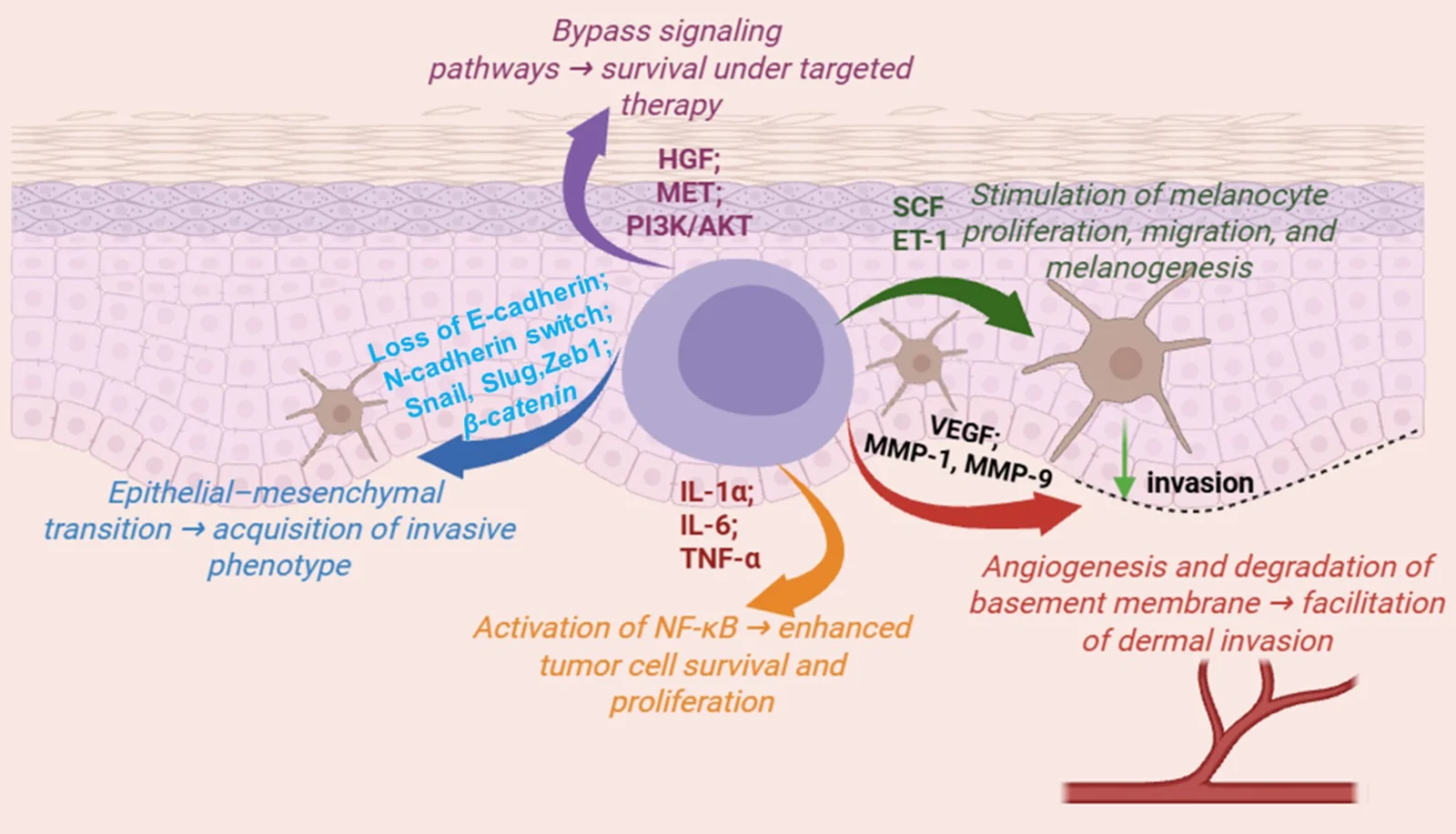
Functional roles of keratinocytes in the melanoma microenvironment.

Keratinocytes also contribute to drug resistance in melanoma. Thus, during targeted therapy with BRAF/MEK inhibitors, keratinocytes secrete hepatocyte growth factor (HGF) and other ligands that activate bypass signaling pathways (MET, PI3K/AKT) in melanoma cells, enabling them to survive in the presence of the anticancer agent [59]. The diverse and partially overlapping mechanisms by which keratinocytes influence melanoma progression are summarized in Figure 1.

Keratinocytes contribute to melanoma progression through five principal mechanisms: (1) regulation of the epidermal niche via SCF and ET-1; (2) inflammatory signaling through IL-1α, IL-6, and TNF-α; (3) promotion of EMT via loss of E-cadherin and cadherin switch; (4) angiogenesis and matrix remodeling through VEGF and MMPs, which degrade the basement membrane and facilitate dermal invasion; and (5) therapeutic resistance via HGF-mediated activation of MET and PI3K/AKT. Keratinocytes do not initiate melanoma but contribute to the formation of a permissive niche that facilitates tumor progression.

Thus, keratinocytes are not merely bystanders but active “regulators” of the clinical course of melanoma. The likelihood of progression depends on their ability to maintain control at the early stage, while their reprogramming during the vertical growth phase provides the tumor with nourishment, invasion, and protection from anticancer agents. However, as the tumor invades and progresses, the center of regulation shifts from the epidermal compartment to the dermal compartment: into the zone of active stromal remodeling, where fibroblasts and immune cells play the key role.

## 3. Cancer-Associated Fibroblast: Orchestrating the Tumor Process

As melanoma invades the dermis, fibroblasts acquiring the cancer-associated fibroblast (CAF) phenotype begin to play a leading role in the TME, shaping the stromal architecture and actively influencing tumor cell behavior [60]. CAFs are characterized by a specific pro-tumorigenic phenotype marked by increased contractility, extracellular matrix production, and secretion of cytokines and chemokines [61]. Among these, the CXC-motif chemokine ligand 12 (CXCL12), also known as stromal cell-derived factor-1, is of particular importance. By interacting with chemokine receptor 4 (CXCR4) expressed on melanoma cells, CXCL12 stimulates their chemotaxis, survival, and migration to pre-metastatic niches, primarily the lungs and lymph nodes [62,63]. Activated CAFs produce a complex of pro-angiogenic and pro-tumorigenic factors—HGF, VEGF, and FGF2—which not only promote vascular network formation but also support melanoma cell survival through activation of the corresponding signaling cascades [61,64,65]. By producing a set of MMPs that degrade the extracellular matrix, CAFs drive ECM remodeling and facilitate melanoma invasion. High MMP expression in melanoma lesions correlates with disease progression and poorer patient survival [66].

CAFs are not a homogeneous population but rather a heterogeneous collection of subtypes with distinct origins, marker profiles, and functions [60]. Single-cell and spatial profiling of melanoma and other skin cancers has delineated several major CAF states: myofibroblastic CAFs (myCAFs), defined by high α-smooth muscle actin expression, increased contractility, and abundant extracellular matrix (ECM) production; inflammatory CAFs (iCAFs), which secrete cytokines and chemokines such as IL-6, CXCL12, and CCL5; and antigen-presenting CAFs (apCAFs), which express MHC class II molecules [60,61]. The relative abundance of these subtypes varies between tumors, between anatomical sites, and over the course of disease, and this heterogeneity translates into distinct, and at times opposing, effects on tumor immunity. Importantly, CAF states are plastic and can interconvert in response to tumor-derived signals: TGF-β drives the myCAF program, whereas IL-1β and IL-6 favor the iCAF program [60,61,64]. This phenotypic flexibility allows the stromal compartment to adapt to, and to reinforce, the immunosuppressive pressures of the TME.

CAFs also actively interact with immune cells in the TME. CAFs promote the migration of Treg cells into the tumor through the secretion of CCL5 or CXCL12, thereby expanding this cell population [67]. In melanoma, CAFs have been shown to promote the formation of so-called “immune exclusion,” in which CD8+ T cells accumulate at the tumor periphery but fail to penetrate its central region, the “tumor core.” This process is largely mediated by the production of CXCL12, TGF-β, and IL-6, as well as the synthesis of a dense fibronectin and collagen matrix [68]. Several complementary mechanisms converge on this outcome. First, by depositing and cross-linking collagen I, fibronectin, and other matrix components, CAFs create a dense, stiff stromal barrier that physically restricts the penetration of CD8+ T cells into the tumor core and limits the diffusion of therapeutic antibodies [68]. Second, the ECM acts as a chemokine reservoir: CXCL12 binds to matrix glycosaminoglycans and is retained at the tumor margin, establishing a local gradient that traps CXCR4-expressing T cells in the stroma instead of guiding them into the tumor parenchyma, while the same CXCL12/CXCR4 axis recruits immunosuppressive populations such as Tregs and myeloid-derived suppressor cells [62,67]. Third, TGF-β, abundantly produced by myCAFs, suppresses the effector functions of CD8+ T cells and natural killer cells, promotes Treg differentiation, and downregulates antigen-presentation machinery, while simultaneously inducing further myofibroblast differentiation and ECM deposition in a self-amplifying loop [64,68]. IL-6 and CCL5 secreted by iCAFs further expand Treg and MDSC populations, consolidating the immunosuppressive niche [67]. Collectively, these mechanisms convert the tumor stroma into a barrier that excludes effector cells and underlies resistance to immune checkpoint inhibitors: tumors with a CAF-rich, T-cell-excluded phenotype respond poorly to anti-PD-1 and anti-CTLA-4 therapy, making CAFs a potential target for combination therapy [64,68,69]. CAFs also contribute to resistance to targeted therapy, as stromal HGF, VEGF, and FGF2 activate complementary signaling pathways in melanoma cells that bypass BRAF/MEK inhibition [59,61,63]. These observations have made CAFs and their products attractive targets for stroma-normalizing combination strategies aimed at restoring T-cell access to the tumor core.

The immune component of the TME includes elements of innate and adaptive cellular immunity, as well as cytokines, chemokines, and other signaling molecules [67]. Studies of various cancer types have shown that the immunological composition of immune cell infiltration—particularly cytotoxic T cells, natural killer cells, dendritic cells, and macrophages—within the TME can predict clinical outcomes and therapeutic responses to anticancer agents [68,69].

## 4. The Immune Component of the Melanoma TME

### 4.1. Granulocytes

Granulocytes comprise neutrophils [70] eosinophils, and basophils, all of which perform a range of protective functions, including phagocytosis, cytokine-mediated pro-inflammatory tissue activation, and antimicrobial defense. Among these, neutrophils are the most abundant and exhibit functional plasticity in the context of cancer [71]: they can promote inflammation through extracellular matrix remodeling or, conversely, exert immunosuppressive effects via the formation of neutrophil extracellular traps and the induction of NETosis, a specialized form of cell death [72].

Under normal conditions, neutrophil adaptation is determined by increasing transcriptional noise in the bloodstream and the influence of local tissue signals that direct the formation of specialized phenotypes in different organs. In cancer, neutrophil maturation is accelerated and promotes the emergence of “transcriptional noise” in tissues, which is accompanied by granulopoietic stress. In the TME, immature neutrophils are released into the blood and rapidly differentiate into pro-angiogenic or antigen-presenting populations [70].

With regard to melanoma specifically, the role of eosinophils in the development and metastasis of this tumor type is frequently highlighted. The presence of tissue eosinophils in nevi or melanomas is associated with a more favorable outcome in patients undergoing immunotherapy [73]. An important prognostic marker is eosinophil cationic protein [74] —its elevated serum level has negative prognostic value, while being simple to use in clinical practice and independent of lactate dehydrogenase levels. Eosinophilia at any stage of the disease correlates with a trend toward longer survival, and in patients receiving immune checkpoint inhibitor therapy, this association becomes statistically significant. Thus, in patients with eosinophil levels above 20%, median survival reaches 35 months—nearly twice the value observed in the group without marked eosinophilia [75]. Moreover, immunotherapy is often accompanied by an increase in the proportion of eosinophils, further underscoring their potential significance in treatment response [73].

Basophils have been linked to IL-4 production in sentinel lymph nodes and tumor lesions in melanoma, with basophil numbers increasing with disease stage and correlating with an unfavorable prognosis [76]. IL-4 promotes Th2 activation and subsequent metastasis. In an analysis of single nucleotide polymorphisms in granulocytes from melanoma patients, significant changes were identified only for neutrophils and eosinophils; basophils did not emerge as a prognostic marker in this setting [77].

### 4.2. Macrophages

Macrophages are representatives of innate immunity that perform multiple functions during inflammation and play an important role in combating tumor cells through phagocytosis and cytokine production [78]. Tumor-associated macrophages (TAMs) possess the ability to alter their phenotype and functional activity in response to the microenvironment [79]. This leads to the formation of distinct subpopulations: activated macrophages are classified into pro-inflammatory M1-like cells and anti-inflammatory M2-like cells [80]. M1-like macrophages exert potent antitumor activity by secreting pro-inflammatory cytokines, including TNF-α, IL-1, and IL-6, as well as cytotoxic mediators such as reactive oxygen species (ROS) and nitric oxide (NO), which directly eliminate tumor cells. Moreover, M1-like macrophages can function as antigen-presenting cells, engaging T cells and recruiting additional immune effectors to the tumor site [81]. In contrast, M2-like macrophages display an anti-inflammatory phenotype and actively support tumor growth, angiogenesis, and immunosuppression. They secrete IL-10 and TGF-β, which suppress the effector functions of T cells and NK cells, thereby facilitating immune escape [82,83,84]. M2-like macrophages also upregulate genes encoding matrix metalloproteinases (MMP-2, MMP-9) and vascular endothelial growth factor A (VEGFA), which promote extracellular matrix remodeling, tumor progression, and metastasis [85]. In melanoma, tumor-associated macrophages predominantly adopt the M2-like phenotype [86], a feature closely linked to immune evasion and poor clinical outcomes. Tumor cells actively recruit circulating monocytes and drive their differentiation into M2-like macrophages through the secretion of factors such as CCL2 and CSF1 [87]. In addition, macrophage migration inhibitory factor (MIF), produced by macrophages, has emerged as a significant regulator of alternative macrophage activation in melanoma models [88].

Suppression of immunosuppressive MIF–CD74 signaling in macrophages and dendritic cells through immunotherapy may restore the antitumor immune response in metastatic melanoma [89]. Furthermore, TME factors, such as hypoxia, promote macrophage polarization toward the M2-like phenotype. Several studies are aimed at repolarizing tumor macrophages from the M2-like to the M1-like phenotype, using target proteins such as TRAF3/STAT6 [90], TLR4 [91,92], iNOS [93], IL-9 [94] and others. The combination of immune checkpoint inhibitors with macrophage modulators may represent an effective therapeutic strategy [95]. Additionally, like T cells, macrophages can be targets of PD-1/PD-L1 axis immunotherapy, as they express this receptor [96]. The combination of immune checkpoint inhibitors and modulators of macrophage activity represents a therapeutic strategy for the treatment of melanoma.

In modern research, the classical classification of TAMs is giving way to a functional-spatial classification derived from single-cell sequencing data [97,98]. Ruo-Yu Ma et al. [97] classified a few TAM subtypes characterized by specific gene signatures and functions using single-cell omics, named: interferon-stimulated IFN-TAMs, immune-modulated Reg-TAMs, inflammatory cytokine-enriched Inflam-TAMs, lipid-associated LA-TAMs, pro-angiogenic Angio-TAMs, resident tissue macrophage-like RTM-TAMs, and proliferative TAMs Prolif-TAMs. Their role in the development of various types of cancer is being studied in detail in numerous publications [99,100], including the tumor type under review, melanoma. Factors secreted by melanoma cells can drive macrophage polarization towards a phenotype that supports tumor progression. Macrophages actively modulate the efficacy of current melanoma therapies: they influence responses to monoclonal antibody treatment, contribute to resistance against MAP kinase inhibitors, and are critically involved in the mechanisms of oncolytic virus therapy [101]. Therefore, therapeutic strategies for melanoma should explicitly account for macrophage–tumor interactions.

### 4.3. Natural Killer Cells

Natural Killer Cells (NK cells) are important players in the antitumor response, as they are capable of detecting pathologically altered cells and eliminating them immediately, serving as the principal sentinels in the body [102]. In addition to direct cytotoxicity, NK cells can also act as regulators [103] of the tumor immune microenvironment by producing inflammatory cytokines and, depending on tumor progression, modulating cell-mediated responses to the tumor. They may participate in the immune-excluded phenotype: NK cells recruited to the tumor zone via the CX3CR1 receptor can suppress the infiltration and activity of CD8+ T cells, and their depletion in melanoma tissue promotes tumor regression during therapy [104]. In the TME of cutaneous melanoma, as in several other malignancies, NK cells demonstrate impaired functional activity. One possible cause of this condition is dysregulation of the NKG2D receptor, which plays an important role in controlling cytotoxic functions, cytokine synthesis, and regulation of the expression of other receptors on NK and other lymphocytes [105]. It has been established that defects in NK cell function correlate with tumor progression, and their development during the course of the disease is influenced by both the tumor itself and factors of the local microenvironment [106]. NK cell levels in peripheral blood may serve as a prognostic marker of response to melanoma immunotherapy [107].

In the melanoma TME, NK cell activity is frequently suppressed. This is facilitated, in particular, by cytokines secreted by the tumor itself. High levels of TGF-β1 are associated with suppression of NK cell activity in melanoma patients [108]. In contrast, increased production of interferon-gamma (IFNγ) is linked to the antitumor effect of natural killer cells [109].

Immune checkpoint inhibitors not only restore T cell activity but can also indirectly potentiate NK cell function by reshaping the TME [83,110]. Specifically, blockade of the PD-1/PD-L1 axis releases inhibitory signals that constrain NK cell effector activity, thereby enhancing their antitumor potential. This dual effect positions NK cells as an attractive therapeutic target in melanoma and supports the development of strategies aimed at boosting their function within the immunosuppressive microenvironment [111].

So, NK cells exhibit a dual functionality in melanoma: they mediate direct tumor cell lysis via recognition of stress ligands and the release of perforin and granzymes, while also secreting cytokines (IFN-γ, TNF-α) to enhance cytotoxicity and orchestrate a coordinated immune response through the NK-DC-T cell axis [112]. Simultaneously, certain NK subsets can act as suppressors by modulating immune cell migration via the CX3CR1/CX3CL1 axis [113], and in some models, moderate depletion of regulatory NK subsets paradoxically promotes tumor regression by relieving immune suppression [114,115]. Overall, the prognostic value of NK cells is highly context-dependent, with cytotoxic NK infiltration in the tumor microenvironment often correlating with improved outcomes in cancers like melanoma.

### 4.4. Dendritic Cells

Dendritic cells (DCs) occupy a central position in antitumor immunity, as they are responsible for the initiation and fine-tuning of T cell-mediated responses [116]. Their ability to capture tumor antigens, migrate to draining lymph nodes, and present processed peptides to naïve T cells makes them attractive targets for immunotherapy in melanoma [117,118,119]. A specialized subset of DCs, Langerhans cells, resides in the epidermis and performs distinct sentinel functions. However, within the melanoma TME, the functional competence of DCs is frequently compromised. Tumor-derived factors—most notably IL-10, TGF-β, and VEGF—impair DC maturation, migration, and antigen presentation, ultimately shifting the balance from T cell activation toward immune tolerance [120]. Type 1 conventional dendritic cells (cDC1) are a promising target for cancer immunotherapy [121]; in melanoma lesions, cDC1 are co-localized with CD8+ T cells, and this is associated with response to immune checkpoint inhibitor therapy. DCs, like other immune cells, have been modified using CAR technology: CAR-DCs suppressed the growth of B16.HER2 tumors, stimulated the proliferation and activation of tumor-infiltrating CD8+ T cells, and, in combination with a PD-1 inhibitor and granulocyte-macrophage colony-stimulating factor expression, provided an enhanced and more durable antitumor response, supporting the potential of CAR-DCs as a promising therapeutic strategy for solid tumors [122]. In addition to CAR, DCs and their precursors have been modified with EVIR receptors directed at tumor recognition, including melanoma [123]. Another approach involves personalized dendritic cell-based vaccines, which can substantially expand the diversity and enhance the activity of tumor-specific CD8+ T cells in melanoma, reshaping antitumor immunity at the molecular level [124]. Evidence indicates that combining DC vaccines with immune checkpoint inhibitors is a promising approach for overcoming T cell exhaustion and improving therapeutic efficacy [125,126].

### 4.5. T-Lymphocytes

Cytotoxic T lymphocytes (CD8+ T cells, CTLs) serve as the key effector arm of the immune response against tumor cells [127]. Their cytotoxic activity is mediated through the recognition of tumor-specific antigens presented by major histocompatibility complex class I (MHC I/HLA I) molecules on the surface of melanoma cells [128]. Binding of the T cell receptor (TCR) to the peptide–HLA I complex triggers target cell killing: CTLs release perforin and granzymes, which induce cell lysis [129], and secrete pro-inflammatory cytokines—interferon-γ (IFN-γ) [130] and tumor necrosis factor-α (TNF-α) [131]—that promote tumor cell apoptosis. In parallel, T helper cells (CD4+ T cells) enhance the antitumor response through cytokine production (including IL-2) [132], stimulating the activation, proliferation, and differentiation of CTLs.

Despite the considerable potential of T cell immunity, melanoma actively suppresses it through a number of mechanisms. A key role is played by the interaction of PD-1 [133] (on T cells) with PD-L1 (on tumor cells) [134], leading to inhibition of T cell activation and the development of an exhausted phenotype [135]. Additional immunosuppressive factors include regulatory T cells (Tregs), which secrete immunosuppressive cytokines (TGF-β, IL-10), as well as adverse TME conditions—hypoxia and low pH—that negatively affect T cell infiltration and functionality [136]. In particular, in melanoma, an increase in Treg numbers is observed in peripheral blood, lymph nodes, and directly within the TME [137], which correlates with reduced cytolytic activity of antitumor immune cells.

Currently, the use of immune checkpoint inhibitors (e.g., pembrolizumab [138,139,140] and nivolumab [141,142]), is widespread; these agents block the PD-1/PD-L1 signaling pathway, restoring T cell functional activity and improving survival in melanoma patients. It has been established that patients who respond to immune checkpoint inhibitors exhibit increased functional activity and abundance of tumor-resident T cells (CD8+ and CD4+ TR), whereas resistance is associated with predominant T cell exhaustion [143,144]. Of particular interest is a spatially organized triad composed of CD8+ TR cells, CD4+ TR cells, and DC3 dendritic cells [145]. Their presence correlates with effective antitumor immunity and may potentially serve as a basis for the development of spatial biomarkers of response to immunotherapy.

### 4.6. B-Lymphocytes

Increasing evidence indicates that tumor-infiltrating B cells play an important role in melanoma development [146,147]. In melanoma, the frequency of B cells is increased compared with normal skin [148], and local B cell proliferation and antibody maturation are observed [149]. Some clinical studies show that elevated B cell content is associated with better survivability [150] but worse outcomes of BRAF/MEK therapy [151], while preclinical studies indicate that B cells may also contribute to the formation of antitumor immunity; thus, their role is not unequivocally positive or negative. Melanoma resistance to BRAF and MEK inhibitors is mediated by a mechanism involving tumor-associated B cells [151], which, under the influence of FGF-2, produce IGF-1 that promotes the emergence of resistant tumor subpopulations; B cell depletion with anti-CD20 antibodies demonstrates antitumor activity. In a study by Harris et al. [152], melanoma lesions were dominated by B cells expressing cytokines (TGF-β, PD-L1) and tending toward regulatory phenotypes [153]. TGF-β-expressing B cells formed clusters and interacted with T cells via CXCL13, CCL4, and CD74 molecules, and with Tregs via CD47–SIRP-γ molecules.

Several studies have yielded contradictory results regarding the activation of these cells in the melanoma TME [154]. Recent melanoma studies have shown that melanoma is characterized by high B cell density, which correlates with a more favorable prognosis [155]. In contrast, in patients with a predominance of B cells, high B cell infiltration of tumor tissue was observed, which corresponded to reduced MAPK kinase activity and increased expression of immunosuppressive markers. Nevertheless, it should be concluded that B cells play a dual role in melanoma: they either support antitumor immunity or promote immune escape.

Most likely, the functional orientation of B cells is determined by their subpopulation composition and the stage of the tumor process: regulatory B cells (Bregs) predominate at late stages, whereas antibody-producing and antigen-presenting subpopulations predominate at early stages, under conditions of active immune surveillance.

B cells exhibit dual anti- and pro-tumoral roles: they support anti-tumor immunity via TLS and activated subsets (e.g., TIPB) that promote organized immune responses and therapy response [156], while regulatory subsets (Bregs), PD-L1 expression, IgG4 shift, and IGF-1-mediated mechanisms can drive immunosuppression and treatment resistance [157]. Then their function is highly context-dependent, varying with tumor type, stage, microenvironment, and therapy.

Immune editing underlies the ability of melanoma to evade immune surveillance and is realized through sequential phases, from elimination to escape [158]; studying the mechanisms of such evasion and the role of danger signals (including programmed cell death pathways) may reveal novel targets for immunotherapy and expand the spectrum of tumors sensitive to treatment [159]. The key mechanisms of escape include defective recognition of tumor antigens due to mutations, resistance of melanoma cells to apoptosis, and the establishment of an immunosuppressive microenvironment: this is maintained by the production of cytokines (TGF-β, IL-10), activation of metabolic pathways (IDO, the CD39/CD73 axis) [160], and overexpression of immune checkpoints. Soluble factors (VEGF, TNF, IL-1, IL-6, PGE2, Fas/Fas-L) [161,162,163], play a substantial role, not only directly suppressing immune cell activity but also impairing dendritic cell maturation and promoting the recruitment of TAMs, MDSCs, and Tregs.

Progressive immune exhaustion is a critical component of melanoma escape: the high proliferative activity of the tumor depletes the pool of effector cells, primarily CD4+ and CD8+ T lymphocytes [164]. In this context, the accumulation of regulatory T cells (Tregs) in the TME represents one of the principal mechanisms of immunosuppression; the CD8+ T cell to Treg ratio is regarded as a prognostic marker of survival in melanoma patients, and modulation of Treg activity is considered a promising direction for enhancing antitumor immunity.

## 5. Prospects for Cellular Technologies in Melanoma Therapy

A deeper understanding of the cellular composition and mechanisms of immunosuppression within the melanoma microenvironment has provided the foundation for the development of a fundamentally new direction—cellular immunotherapy, which involves the use of living cells, their modified variants, or their derivatives to restore or enhance the antitumor immune response [165]. Despite a large number of preclinical studies, the number of completed randomized phase II and III clinical trials in the field of cellular immunotherapy for melanoma remains limited, which complicates the definitive assessment of its clinical efficacy. The key completed phase II–III clinical trials reflecting the main directions of cellular immunotherapy for melanoma are presented in Table 2. In patients with refractory melanoma, improved outcomes have been achieved through adoptive therapy using tumor-infiltrating lymphocytes (TILs) expanded ex vivo, or T cells genetically modified with T cell receptors (TCRs) specific for melanoma antigens [166].

The most impressive results in cellular therapy for melanoma have been demonstrated by TIL therapy. This autologous approach involves resection of a metastatic tumor lesion, isolation of lymphocytes from it, their in vitro expansion with IL-2, and subsequent infusion into the patient after non-myeloablative lymphodepletion. A phase II study led by Rosenberg S.A. and Goff S.L. showed that in patients who had previously progressed on immune checkpoint inhibitors, objective responses were achieved in 36–40% of cases, with complete regression observed in 10–15% of patients [169]. In addition, modified TCR therapies targeting melanoma antigens (such as gp100) are being investigated, and TCR-based therapy targeting MAGE-A4 (afamitresgene autoleucel [afami-cel]) is under development [170].

If approved, TIL therapy may become a treatment option after failure of immunotherapy and targeted therapy. TIL therapy in patients with metastatic melanoma has demonstrated significant response rates of ≥50%, with complete remission observed in 22% of patients [171].

Lifileucel (LN-144) is an autologous TIL product administered as a single infusion. It is manufactured through a centralized 22-day process that expands patient-derived tumor T cells into a heterogeneous population of billions of polyclonal CD4+ and CD8+ lymphocytes capable of recognizing multiple tumor-associated neoantigens [172]. Following a single infusion of lifileucel, the T lymphocytes (a mixture of CD4 and CD8 cells) migrate to tumor sites throughout the body, where they recognize and target various individual tumor-associated neoantigens and mediate tumor cell lysis [172]. Lifileucel has demonstrated clinically meaningful activity in patients with advanced melanoma and high tumor burden [173]. While the development of lifileucel was primarily aimed at centralizing and optimizing TIL manufacturing, KSQ Therapeutics and Instil Bio focused on enhancing their antitumor activity. In particular, KSQ Therapeutics employed CRISPR/Cas9-based functional genomic screening to identify therapeutically relevant targets in immune cells. Primary screening of T cells conducted in a mouse model identified the CT-1 (Cell Therapy-1) target with clinical potential. Further studies in C57BL/6J mice bearing B16-Ova melanoma showed that TCR-Tg T cells with CT-1 knockout were 10-fold more effective than control-edited cells. In a second screen performed on human T lymphocytes, CT-1 knockout also ranked among the most significant factors enhancing cell expansion. These findings formed the basis for the development of KSQ-001—genetically modified TILs (eTILs) in which the CT-1 gene was edited using CRISPR/Cas9. In preclinical models, KSQ-001 demonstrated increased secretion of interferon-γ and TNF-α, as well as elevated expression of genes associated with antitumor activity, compared with control edited cells [174].

One of the key limitations of TIL therapy is its high logistical complexity, due to the need for fresh tumor material, which can be obtained in only 60–70% of patients with metastatic melanoma. Moreover, the lengthy manufacturing process (4–6 weeks) may be critical for patients with rapidly progressing disease [175].

Whereas TIL therapy depends on the successful isolation and amplification of T cells from tumor tissue and the presence of melanoma-specific TCRs [166], chimeric antigen receptor (CAR) T cells are manufactured ex vivo from circulating blood T cells [165]. Through their extracellular antigen-binding domain, CAR-T cells interact with a tumor-associated surface protein in an MHC-independent manner, leading to T cell activation and subsequent antigen-specific killing of tumor cells [176]. In recent years, several CARs targeting melanoma cells have been developed in preclinical studies, including those specific for chondroitin sulfate proteoglycan 4 (CSPG4) or tyrosinase-related protein 1 (TYRP1) [177]. CAR-T cell therapy against integrin αvβ3 has demonstrated high efficacy in eliminating malignant cells in preclinical models of melanoma and other solid tumors [178]. A phase I clinical trial demonstrated the safety and feasibility of intravenous CAR-T cell therapy directed against c-MET in patients with metastatic melanoma (NCT03060356) [179].

A key obstacle to CAR-T cell activity is the immunosuppressive TME, mediated by myeloid-derived suppressor cells (MDSCs), regulatory T cells (Tregs), macrophages, and other components of the TME [180] as well as the density of the TME [181].

Dendritic cell (DC)-based immunotherapy exploits the unique ability of DCs to capture, process, and present tumor-associated antigens to T lymphocytes, thereby initiating potent adaptive immune responses against melanoma [182]. Approaches include the generation of autologous DCs ex vivo from patients’ peripheral blood monocytes, followed by loading with antigens—peptides, tumor lysates, or messenger RNA encoding neoantigens. After reinfusion into patients, fully mature DCs migrate to lymphoid tissues, activating CD4+ helper and CD8+ cytotoxic T cells, resulting in targeted lysis of tumor cells.

A number of phase I–III clinical trials have focused on the activation of plasmacytoid DCs (pDCs) in the tumor through the use of TLR7 and TLR9 agonists, which reactivate the production of type I interferons and cytotoxic molecules (e.g., TRAIL) [183]. A clinical trial involving subcutaneous injection of TLR9 ligands in patients with advanced melanoma demonstrated pDC activation and partial clinical responses or disease stabilization in 25% of patients [184]. The use of imiquimod (IMQ, a TLR7 agonist) in a humanized mouse model of melanoma suppressed tumor progression after pDC mobilization and induction of their cytotoxic functions [185]. In a randomized phase 3 trial [186], patients with resected high-risk stage IIIB and IIIC melanoma received treatment with dendritic cell nanoparticles (nDCs) or placebo. In the nDC treatment group, 67.1% of patients developed a functional T cell response against the antigens used to load the nDCs. Unfortunately, these immunological responses did not translate into clinical benefit. The study did not meet its primary endpoint, and the curves even show a slight, statistically non-significant decrease in survival in the nDC treatment group.

Macrophages, as integral elements of the TME, perform a dual role: as innate immune cells and as antigen-presenting cells. Furthermore, their long half-life in the circulation is a significant advantage [187]. In a recent study, macrophages were engineered to continuously maintain a pro-inflammatory phenotype within the TME. Specifically, the researchers used lentiviral transfection to create genetically modified macrophages expressing TRAIL, a promising antitumor cytokine known to induce apoptosis in tumor cells [188]. This modification enabled the macrophages to sustain their pro-inflammatory phenotype in the TME. In vitro co-culture systems showed that these genetically modified macrophages significantly inhibited melanoma cell growth [189].

Similar to CAR-T cell therapy, CAR-macrophages are created through genetic engineering to express receptors that specifically target tumor-associated antigens. This modification allows them to precisely identify, attach to, and destroy cancer cells [190]. CAR-macrophages demonstrate great potential for targeting solid tumors because they can penetrate the dense TME [191]—a challenge frequently encountered by CAR-T cells due to physical barriers such as the extracellular matrix [181]. In a recent study, CAR-macrophages targeting chondroitin sulfate proteoglycan 4 (CSPG4), which is abundantly expressed on melanoma cells, were developed and demonstrated the ability to selectively phagocytose CSPG4-expressing melanoma cells. In mouse models, these CAR-macrophages effectively suppressed melanoma tumor growth. Furthermore, the combination of CSPG4-targeted CAR-macrophages with CD47-blocking antibodies synergistically enhanced phagocytic activity and significantly inhibited the growth of melanoma spheroids in 3D cultures [192].

### Key Barriers to Cellular Therapy in Melanoma

Despite encouraging results in selected patients, cellular therapy in melanoma and other solid tumors faces several fundamental barriers. First, antigen heterogeneity limits the efficacy of single-antigen-targeted approaches: melanoma tumors frequently display heterogeneous expression of target antigens, antigen loss, and clonal evolution, enabling immune escape and reducing the durability of responses. Second, poor trafficking and infiltration of adoptively transferred cells into tumor tissue remain critical obstacles, largely due to abnormal tumor vasculature, dense extracellular matrix, and chemokine mismatch. Third, the immunosuppressive TME—characterized by TGF-β, IL-10, adenosine, regulatory T cells, myeloid-derived suppressor cells, and M2-like macrophages—actively suppresses effector cell function and limits the persistence of transferred cells. Fourth, metabolic barriers, including hypoxia, glucose deprivation, lactate accumulation, and adenosine signaling, further impair the function and survival of T cells and NK cells within the tumor. These limitations have motivated the development of next-generation platforms such as armored CAR-T cells, cytokine-secreting constructs, and metabolically reprogrammed cellular products, as well as combination strategies aimed at normalizing the TME.

Given these barriers, the choice of cellular platform requires a personalized approach. TIL therapy demonstrates the most mature clinical results but is constrained by logistical complexity and dependence on the availability of tumor material, whereas CAR-T cells manufactured from peripheral blood face the immunosuppressive microenvironment and the density of the stroma of solid tumors. In this context, CAR-macrophages—capable of penetrating dense tumor stroma, phagocytosing tumor cells in an MHC-independent manner, and maintaining a pro-inflammatory phenotype—are of particular interest as a therapeutic platform. The negative results of randomized trials of dendritic cell vaccines indicate the insufficiency of isolated activation of the T cell compartment and underscore the need for comprehensive modulation of the TME. The most well-founded strategy for the further development of cellular therapy for melanoma appears to be the combination of cellular platforms with immune checkpoint inhibitors, stromal modulators, and macrophage-repolarizing agents, as well as the implementation of personalized approaches that take into account the individual composition of the TME.

## 6. Conclusions

The analysis presented herein allows the melanoma TME to be viewed not as a collection of individual cell populations, but as a unified immunosuppressive network in which keratinocytes, cancer-associated fibroblasts, and immune cells are functionally interconnected. The reprogramming of keratinocytes, the establishment of CAF-mediated “immune exclusion,” the predominance of M2-phenotype macrophages, the expansion of regulatory T and B lymphocytes, the accumulation of MDSCs, and the suppression of NK cell and dendritic cell activity collectively create a self-sustaining circuit of immunosuppression. It is this circuit, rather than the intrinsic properties of tumor cells alone, that determines the limited efficacy of current therapeutic approaches and dictates the need for strategies aimed at disrupting it.

To date, many researchers agree that targeting the microenvironment has become the key concept of modern cellular immunotherapy for melanoma. The greatest clinical success has been achieved in the field of TIL therapy; however, its broad application is constrained by logistical complexity and dependence on the availability of tumor material. Alternative platforms—CAR-T cells and CAR-macrophages—have demonstrated encouraging results but face the challenges of the immunosuppressive microenvironment and stromal density. In this context, CAR-macrophages are of particular interest, as they are capable of penetrating dense stroma, phagocytosing tumor cells in an MHC-independent manner, and maintaining a pro-inflammatory phenotype.

Personalized assessment of the TME composition, including the spatial distribution and functional state of immune cells, may serve as the basis for patient stratification and the selection of the optimal cellular platform. It is also worth noting that PRAME, a shared antigen between melanoma and testicular germ cell tumors, has become a target of TCR-T therapy (IMA203), currently in a phase 3 trial. This cross-tumor parallelism suggests that reprogramming strategies developed for germ cell tumors may inform the development of cellular therapies for melanoma. Given the persistent problem of resistance to standard treatment modalities, cellular immunotherapy aimed at “reprogramming” the melanoma microenvironment represents an actively investigated and rapidly evolving direction in oncoimmunology.

## Figures and Tables

**Table 1:** Factors Determining Sex-Based Heterogeneity in Melanoma Prevalence and Clinical Course.

Men	Women	Clinical Significance
**Behavioral** **and Anatomical Features**		
Less frequent use of photoprotection; melanoma is more commonly localized to the trunk, head, and neck (areas of chronic/intense UV exposure) [21].	More frequent use of sunscreens; melanoma is more commonly localized to the lower extremities (areas of intermittent UV exposure) [5,22].	Hidden localizations in men may contribute to later detection, although direct evidence for diagnostic delay is limited [23]. The potential value of sex-specific screening (in men—thorough examination of the scalp, ears, and back; in women—emphasis on the lower extremities) has been proposed but is not currently established as a clinical recommendation.
**Genetic and Epigenetic Features**		
NRAS and TP53 mutations, associated with a more aggressive clinical course, are more frequently detected in men. Higher tumor mutational burden (TMB); accelerated telomere shortening correlates with poorer prognosis and metastasis [24].	Longer telomere length is associated with an increased risk of melanoma [25]; BRAF mutations have been reported more frequently in young women in some cohorts, although this association is not consistently observed across studies [26].	Emerging evidence suggests that TMB may serve as a marker of response to immunotherapy in men, whereas BRAF mutational status in women may guide the choice of targeted therapy (BRAF/MEK inhibitors) [27]. These observations require prospective validation before informing clinical decision-making
**Immunological Dimorphism**		
Preclinical studies suggest that androgens may suppress T-cell activation and promote MDSC expansion in the TME. Clinical data remain limited. An increased frequency of T cell exhaustion has been described, along with a trend toward poorer response to monotherapy with immune checkpoint inhibitors [28,29].	Genes located on the X chromosome that escape inactivation (TLR7, FOXP3, CD40L) may provide women with a “double dose” of key immunoregulatory proteins. Estrogens may enhance interferon-γ production and the cytolytic activity of CD8+ T cells. A stronger innate and adaptive immune response and a trend toward better response to anti-PD-1 therapy in premenopausal women have been reported [28,30]	A hypothesis is emerging regarding potential sex-based stratification of immunotherapy. However, sex-based selection of treatment regimens is not currently recommended in clinical practice. Women may be at higher risk of immune-related adverse events [31].

**Table 2:** Key Completed Phase II–III Clinical Trials Evaluating Cellular Immunotherapy Platforms in Melanoma.

Trial ID	Strategy/Agent	Target	Study Design	Phase	*N*	Key Outcomes	Status	Ref.
NCT02278887	TIL therapy (lymphodepletion + high-dose IL-2)	Multiple tumor neoantigens	Randomized vs. ipilimumab	III	168	mPFS 7.2 vs. 3.1 mo (HR 0.50; *p* < 0.001); ORR 49% vs. 21%; mOS 25.8 vs. 18.9 mo	Completed	[165]
NCT06743126	TCR-T (PRAME-specific)	PRAME	Single-arm	I/II	Several dozen	ORR 50–67% in high-dose cohorts; durable responses in anti-PD-1–resistant patients	Investigational	[167]
NCT02360579	Lifileucel (LN-144)	Multiple tumor neoantigens	Single-arm	II (registrational)	153	ORR 31.4%; DCR 77.8%; mDOR not reached	FDA approved (2024)	[168]

## Data Availability

No new data were created or analyzed in this study. Data sharing is not applicable to this article.

## Abbreviations

The following abbreviations are used in this manuscript:

CAF	Cancer-associated fibroblast
CAR	Chimeric antigen receptor
CTL	Cytotoxic T lymphocyte
DC	Dendritic cell
IFN-γ	Interferon gamma
IL	Interleukin
MAPK	Mitogen-activated protein kinase
MDSC	Myeloid-derived suppressor cell
MHC	Major histocompatibility complex
MMP	Matrix metalloproteinase
NK	Natural killer (cell)
PD-1	Programmed cell death protein 1
PD-L1	Programmed death-ligand 1
TGF-β	Transforming growth factor beta
TIL	Tumor-infiltrating lymphocyte
TME	Tumor microenvironment
Treg	Regulatory T cell
VEGF	Vascular endothelial growth factor
